# Basal Cell Carcinoma: Pathology, Current Clinical Treatment, and Potential Use of Lipid Nanoparticles

**DOI:** 10.3390/cancers14112778

**Published:** 2022-06-03

**Authors:** Izabela Łasińska, Aleksandra Zielińska, Jacek Mackiewicz, Eliana B. Souto

**Affiliations:** 1 Department of Medical and Experimental Oncology, Heliodor Święcicki Clinical Hospital, Poznań University of Medical Sciences, 16/18 Grunwaldzka Street, 60-786 Poznań, Poland; jmackiewicz@ump.edu.pl; 2 Department of Nursing, Institute of Health Sciences, University of Zielona Góra, Energetyków Street 2, 65-417 Zielona Góra, Poland; 3 Institute of Human Genetics, Polish Academy of Sciences, Strzeszyńska 32, 60-479 Poznań, Poland; aleksandra.zielinska@igcz.poznan.pl; 4 Department of Diagnostics and Cancer Immunology, Greater Poland Cancer Centre, 15 Garbary Street, 61-866 Poznań, Poland; 5 Department of Pharmaceutical Technology, Faculty of Pharmacy, University of Porto, Rua de Jorge Viterbo Ferreira, nº. 228, 4050-313 Porto, Portugal; ebsouto@ff.up.pt; 6 REQUIMTE/UCIBIO, Faculty of Pharmacy, University of Porto, Rua de Jorge Viterbo Ferreira, nº. 228, 4050-313 Porto, Portugal

**Keywords:** basal cell carcinoma, solid lipid nanoparticles, nanostructured lipid carriers, liposomes

## Abstract

**Simple Summary:**

Basal cell carcinoma (BCC) belongs to one of the most common types of skin carcinoma characterized by high morbidity worldwide. Available therapies are mainly based on non-targeted approaches, which encounter a significant risk of systemic toxicity in several organs. In this work, we discuss a novel approach using targeted therapy-based lipid nanoparticles loaded with classical chemotherapeutic drugs, Hedgehog inhibitors, or photosensitizers for improved localized therapy while reducing adverse side effects and systemic toxicity. New therapeutic topical strategies are presented despite the limited number of reports available.

**Abstract:**

Skin cancer is the most common type of carcinoma diagnosed worldwide, with significant morbidity and mortality rates among Caucasians, in particular basal cell carcinoma (BCC). The main risk factors of BCC are well-identified, and there are many chemotherapeutic drugs available for its treatment. The effectiveness of therapeutic options is governed by several factors, including the location of the tumor, its size, and the presence of metastases (although rare for BCC). However, available treatments are based on non-targeted approaches, which encounter a significant risk of systemic toxicity in several organs. Site-specific chemotherapy for BCC has been proposed via the loading of anticancer drugs into nanoparticles. Among various types of nanoparticles, in this review, we focus on potential new regimens for the treatment of BCC using classical anticancer drugs loaded into novel lipid nanoparticles. To meet patient aesthetic expectations and enhance the effectiveness of basal cell carcinoma treatment, new therapeutic topical strategies are discussed, despite a limited number of reports available in the literature.

## 1. Introduction

**Figure 1:**
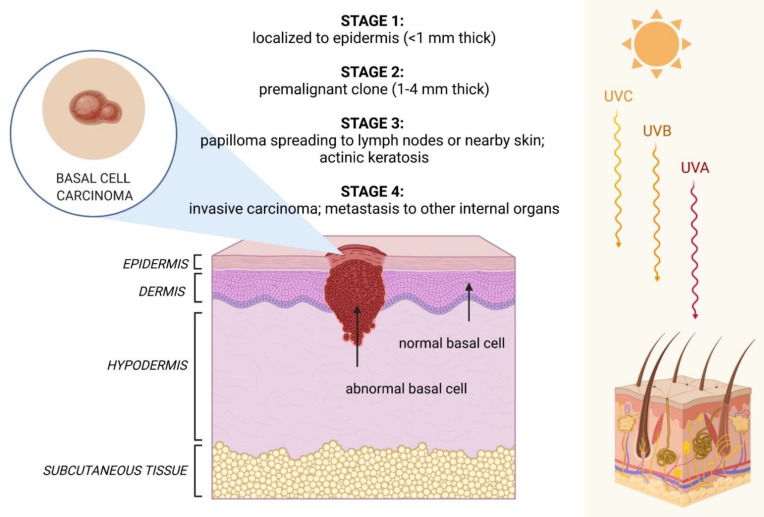
BCC onset related to sun exposure consisting of four stages (I-IV) (own drawing).

Basal cell carcinoma (BCC) is the most common cancer in Caucasians, representing about 85% of non-melanoma skin cancers [1,2]. BCC appears on sun-exposed parts of the body, especially on the skin of the face and on the nose area [3,4]. The main risk factors for BCC development include ultraviolet light (UV) exposure, light skin type, long-term immunosuppression, male sex, advanced age, and certain genodermatoses [5]. The stages of BCC onset related to sun exposure are represented in Figure 1. Familial history of BCC with Gorlin-Goltz syndrome has also been well-described [6]. Several attempts have been made to describe risk scores for BCC and other skin cancers for early detection and radical treatment of disease [7].

The basal cells composing the skin are located in the basal layer of the epidermis, and during maturation, cells migrate to the surface, giving rise to the future epidermis [8,9]. The Hedgehog pathway (HH) plays a crucial role in embryonic development and adult tissue homeostasis. This pathway was first described in *Drosophila* but is also conservative for mammals [10]. In mammals, the HH consists of three secreted ligands: Sonic Hedgehog, Indian Hedgehog, and Desert Hedgehog. There are also transmembrane receptors: Patched 1 (Ptch1), Patched 2, and transmembrane G protein-coupled receptor Smoothened (Smo). Activation of the HH results in stimulation of transcription factors, such as Gli1, Gli2, and Gli3. There is also a suppressor of fused homolog (SUFU), which can modulate Gli activation.

Interestingly, Ptch1 functions as an inhibitor of signal transduction in HH by blocking Smo when the ligand of HH is absent. In sporadic BCC, somatic mutations of *PTCH1* and *SMO* genes are generally described. Additionally, somatic and germline mutations of *SUFU* were discovered [10]. The mutations mentioned above lead to constant activation of HH and uncontrolled canonical cell proliferation.

Diagnosis of Gorlin-Goltz syndrome requires the fulfilment of several criteria. Gorlin-Goltz syndrome is an autosomal dominant inherited syndrome with germline mutations in genes coding HH. Most cases are related to *PTCH1* mutations, which is an HH component. Patients suffer from palmar and plantar pitting, lamellar calcification of the falx cerebri, odontogenic keratocysts of the jaw, rib anomalies, and ocular abnormalities, among other symptoms.

For early detection of BCC, dermatologic examination should start at around 10 years old Radiotherapy treatment is contraindicated because of the increased risk of new BCC lesions in the irradiated area [11,12].

UV light exposure is the leading cause of BCC and other skin cancers. Both UV wavelength A and B are now considered class I carcinogens in humans. This cancerogenic effect depends on the degree of DNA damage due to UV absorption by nuclear DNA and leads to cyclobutane pyrimidine dimers and pyrimidine (6-4) pyrimidone photoproducts. Damaged basal cells exhibit typical UV mutations (transition of C→T or CC→TT). Reactive oxygen species production also increases and may cause T→G transversion [13]. Additionally, UV suppression of adaptive immune response is among the mechanisms that cause skin cancer, although it is not precisely understood [14].

Expression of matrix metalloproteinases, enzymes with the ability to degrade extracellular matrix components, is increased due to UV exposure. Metalloproteinases are responsible for angiogenesis, invasion, and tumor growth and may originate from different sources, including from the tumor microenvironment and from neoplastic cells. Following UV exposure, keratinocytes may secrete many mediators and stimulate the release of metalloproteinases from fibroblasts. Cytokines (e.g., interleukin 1, interleukin 6, interleukin 8, and TNF-α) may be released, activating the mitogen-activated protein kinase pathway (MAPK). MAPK regulates gene expression, cellular growth, and cell survival. Upregulation of MAPK signaling may lead to uncontrolled, increased basal cell proliferation and resistance to apoptosis [15,16,17].

Besides the canonical ligand-Ptch1-Smo route, there are several options for Gli activation independent of Smo. In some types of cancers, both activation routes may coexist. Such cancers are less sensitive to Smo inhibitors. Cross talk between RAS-RAF-MERK-ERK, PI3K-AKT-mTOR, and PTPN14-HIPPO-yap pathways, as well as TGF β signaling with HH, may cause constant activation of Gli [18,19]. Inactivation of tumor suppressor gene *P53* by UV leads to the development of many kinds of cancers, including BCC [20].

As cancer cells are recognized as foreign bodies, cancers are now considered diseases of the immune system. After solid organ transplants, patients must be treated with steroids, resulting in increased risk of neoplasms, including BCC. Furthermore, treatment with an immune checkpoint inhibitor has been proven effective in BCC. Attempts are being made to define risk scores to determine the risk of BCC in patients after organ transplantation [21,22,23].

## 2. Clinical Features of BCC

There are several pathological types of BCC, namely, nodular, superficial, infiltrative, micronodular, and morphea forms. Nodular BCC is the most common subtype. It appears as an area with visible blood vessels or as a raised lump with pearly-white, red, or pink color on top. Superficial BCC appears as a scaly area with pink or red color. Infiltrative and micronodular types can grow deeper into the inner layers of the dermis. These types appear similar to superficial BCC. The morphea form of BCC can look like a scar or white/yellow firm area [24]. Often, the lesion grows very slowly, even for several years, and may be asymptomatic. Only after BCC has reached large dimensions can it cause pain, bleeding, or ulceration. BCC is rarely associated with metastasis and has low mortality rates. However, local infiltration and penetration are possible even deeper into the dermis [25].

There are two main difficulties associated with BCC: disease recurrence, even following radical resection, and field carcinogenesis—the possibility of multiple BCC lesions in nearby UV-damaged skin. Both recurrence and carcinogenesis fields are related to various BCC resections and many scars. Location on the skin of the face is challenging for both patients and surgeons in terms of repeated surgical procedures [26].

## 3. Available Therapies

A new classification for different types of BCC is proposed: ‘easy-to-treat’, which is for common BCC, and ‘difficult-to- treat’, e.g., in the eyelid area. The first type of BCC diagnosis is based on clinical appearance and dermatoscopic features. For the second type, histopathological confirmation is obligatory in BCC located in high-risk, as well as for ambiguous lesions.

Complete surgery is the first-line treatment for ‘easy-to-treat’ BCC lesions [27], that should result in a clear margin via indicated radical resection, to reduce the recurrence risk. However, sometimes, BCC can reappear, even three years after a definitive surgical procedure [28]. Surgery, which is microscopically controlled, may be an option for BCC in critical anatomical sites after recurrence or high-risk lesions. Some topical therapies, such as 5% imiquimod or 5% fluorouracil, are suggested for superficial BCC. The mechanism of action of imiquimod is the stimulation of innate and acquired immune response, leading to inflammatory cell infiltration and apoptosis of BCC within the field of drug application. Fluorouracil is a chemotherapeutic agent, a fluorinated pyrimidine. It interferes with DNA synthesis via thymidylate synthetase inhibition and inhibits BCC proliferation [29,30]. Laser ablation, electrocautery, or electrochemotherapy with bleomycin can also be applied in low-risk superficial BCC [31]. Photodynamic therapy is used in combination with photosensitizing agents, e.g., methyl aminolevulinate. Activation of a drug may take effect only after using light with a certain wavelength, resulting in BCC cells apoptosis, depending on singlet oxygen production, the lowest excited state of the dioxygen molecule [32]. Photodynamic therapy is recommended for the treatment of superficial and thin BCC. Radiotherapy could be an option BCC located on the face for elderly patients who are not suitable candidates for surgery [27].

Therapy for a ‘difficult-to-treat’ BCC should always be discussed by a multidisciplinary team comprising a surgeon, a dermatologist, a clinical oncologist, and a radiotherapist. Close collaboration of specialists may lead to organ preservation, e.g., in the eyelid or nose region.

### 3.1. Treatment of Locally Advanced/Metastatic BCC

Two oral HH inhibitors for first-line treatment have been approved for patients with locally advanced and metastatic disease. Semi-synthetic derivatives of natural alkaloid cyclopamine, which are isolated from *Veratrum californicum*: vismodegib and sonidegib and are small, oral-targeted Smo inhibitors [33,34].

In the Erivance trial, 104 BCC patients with locally advanced and/or metastatic disease were treated with 150 mg vismodegib daily until disease progression or intolerable toxicity. Investigator-assessed objective response rates (ORRs) were 60.3% in the locally advanced and 48.5% in the metastatic BCC group. The median duration of response (DOR) was 26.2 months in the in locally advanced group and 14.8 months in the metastatic group. Median overall survival (OS) was not reached in a locally advanced population of patients and was 33.4 months for metastatic BCC. All patients experienced one or more adverse side effects, regardless of grade, according to the Common Terminology Criteria for Adverse Events (CTCAE). Weight decrease, muscle spasms, and fatigue (grade 3 and 4) were described most often as serious side events. Despite the high efficacy of treatment, 26% of patients withdrew consent, and in 21.2 of patients, treatment was not continued due to side effects [35]. Another trial was conducted with vismodegib in a locally advanced and metastatic setting with a larger group of 1232 BCC patients. The results were consistent with those of the Erivance study, showing a considerable benefit in survival, and ORR and similar toxic profiles as in Gorlin-Goltz syndrome patients [36]. Vismodegib also showed high efficacy [37,38,39].

In the BOLT trial, 230 patients with locally advanced and/or metastatic BCC were treated with sonidegib: 79 with a 200 mg oral daily dose and 151 with an 800 mg oral daily dose. Treatment continued until disease progression or intolerable toxicity. The primary efficacy endpoint was ORR, and according to a central review, ORRs were 56% (95% confidence interval (CI), 43–68) for locally advanced BCC and 8% (95% CI, 02–36) for metastatic BCC in a group of patients with a lower dose of sonidegib. In the group receiving a higher dose of sonidegib, the ORR was 46.5% (95% CI, 37.2–55.1) among locally advanced BCC patients and 17% (95% CI, 5–39) for metastatic BCC. Side effects in the group treated with 200 mg of sonidegib were reported in 43% of patients with grade 3–4 disease according to CTCAE and in 64% of patients in the group treated with a higher dose of sonidegib. The most often reported AEs in grade 3–4 were creatine kinase increase, muscle spasms, and weight decrease. The most common reason for treatment discontinuation was diagnosed side effects: 23 patients (29%) in the group with a lower dose of sonidegib and in 57 patients (37.7%) in the second group [40].

Several parameters were investigated in a meta-analysis, including 18 articles about the treatment of locally advanced/metastatic BCC with HH inhibitors. Vismodegib and sonidegib showed efficacy in a locally advanced stage of disease with similar, overall response rates of 69% and 57%, respectively. However, complete response rates were different: 31% vs. 3%, respectively for vismodegib and sonidegib, respectively. In metastatic BCC, the overall response for vismodegib was 2.7-fold higher than the overall response for sonidegib (39% vs. 15%, respectively). Adverse side effects for both medicines included muscle spasms, dysgeusia, and alopecia [41]. On the one hand, oral drug intake is convenient for patients, but on the other hand, it causes serious side effects, leading to treatment discontinuation. Because HH signaling is crucial in adults for neurophysiological taste sensation, hair follicle development, and follicle bulge stem cell maintenance, identification of topical drugs may reduce the frequency of side effects [42,43]. Vismodegib is approved worldwide for use in patients with unresectable locally advanced/metastatic BCC. Sonidegib is approved for the treatment of unresectable locally advanced BCC and for metastatic BCC, although only in Switzerland and Australia [40].

Although HH inhibitors are effective in treating BCC, there is a possibility of developing drug resistance. Furthermore, primary and secondary HH inhibitor resistance has been described. There are several mechanisms of resistance, e.g., mutations of *SMO* or non-canonical pathway activation due to cross talk. Identifying resistance biomarkers will help in the search for active drugs for second-line treatment [44]. BCC has a very high mutational burden compared to other human malignancies and is therefore more responsive to PD1 blockade [45,46]. Additionally, the risk of BCC is elevated in solid organ transplants recipients, which suggests that adaptive immune responses are essential for disease development [22]. The immunotherapeutic efficacy of anti-PD1 checkpoint inhibitor antibody cemiplimab was examined. Enrolled patients had locally advanced/metastatic BCC and were either intolerant to previous therapy or had no better than stable disease according to Response Evaluation Criteria in Solid Tumors 1.1 (RECIST) after nine months of HH inhibitor treatment. Patients received 350 mg of cemiplimab intravenously every three weeks until progression or intolerable toxicity. Only results for locally advanced BCC (84 patients) were reported, and objective response per independent review was described in 26 patients, with 5 complete responses and 21 patients with partial response. The most common AEs in grades 3–4, according to CTCAE, were hypertension, colitis, fatigue, and urinary tract infection [47].

### 3.2. Aesthetic Expectations of Patients

Extensive, repeated BCC resection, despite its classification as a ‘gold standard of treatment’ with the lowest recurrence rate, often results in social exclusion and reduced self-esteem among patients [48]. New approaches to BCC treatment have been advanced to meet patient needs. Intratumoral remedies, topical use of HH inhibitors, and neoadjuvant indications have been investigated [49].

Owing to patients’ aesthetic expectations, a trial with vismodegib, a previously approved drug for locally advanced/metastatic BCC, was conducted to reduce operational risk after resection for neoadjuvant treatment. In the VISMONEO study, 55 patients with sporadic locally advanced BCC of the skin of the face were enrolled. Vismodegib was administered orally once daily for 4–10 months before planned surgery, achieving the best drug response. As a result, 44 patients had tumor downstaging after vismodegib treatment, representing 80% (95% CI, 67–90) of the enrolled population. Complete response according to RECIST 1.1 was described in 27 of 44 responders, with a median duration of treatment of six months. The overall response rate was 71% (95% CI, 59–88). Adverse side effects were similar to those of HH inhibitor use. Patients had downstaged tumors and a higher probability of a better aesthetical outcome in the face location [50].

Patients with Gorlin-Goltz syndrome and multiple BCC lesions may have many scars after radical resections. A pivotal phase 2 trial of a topical HH inhibitor, patidegib, was conducted in 17 patients. Twice daily use of topical patidegib led to complete response among 25% of patients with existing BCC in the face area (*p* = 0.02) during the six-month treatment period. Additionally, topical use of patidegib caused no taste or hair loss, and patients did not report muscle cramps [51].

## 4. Nanoparticle-Related Therapies for BCC

The skin is an impressive barrier against topical absorption of drugs due to the presence of corneocytes, which are the last stadium of keratinocytes differentiation in the upper layers of the epidermis. Possible routes for nanoparticles permeation via corneocytes are either intracellular or extracellular. Nanoparticles may also permeate the stratum corneum via transappendageal pathways, e.g., through the hair follicles or through sebaceous, pilosebaceous, and sweat glands; however, these cover only 0.1% of the skin surface [52,53,54].

**Figure 2:**
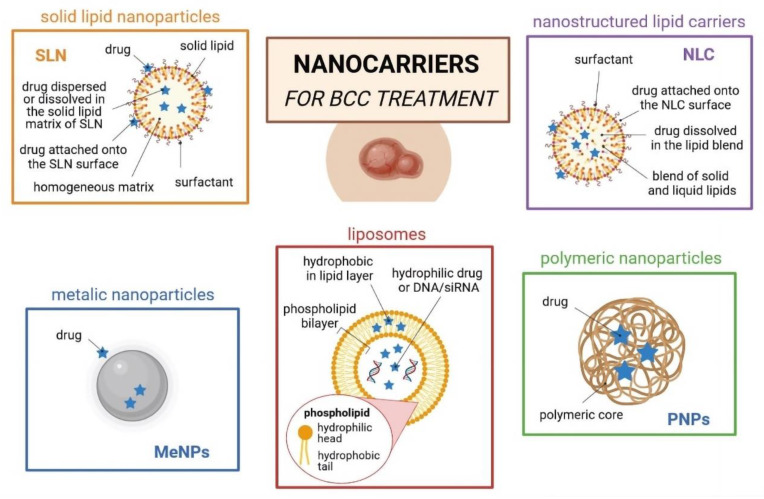
Schematic characterization of the most commonly used nanoparticles for BCC treatment, namely solid lipid nanoparticles (SLNs), nanostructured lipid carriers (NLCs), metal-based nanoparticles, liposomes, and polymeric nanoparticles (own drawing).

**Figure 3:**
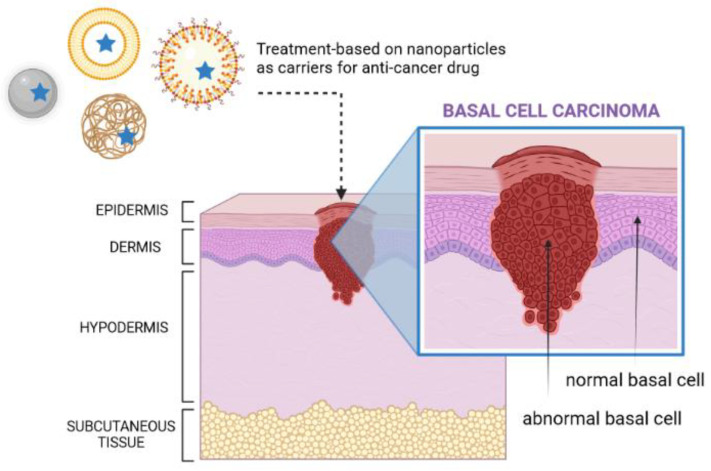
Skin with BCC and different types of nanoparticles loaded with anticancer drugs (own drawing).

**Figure 4:**
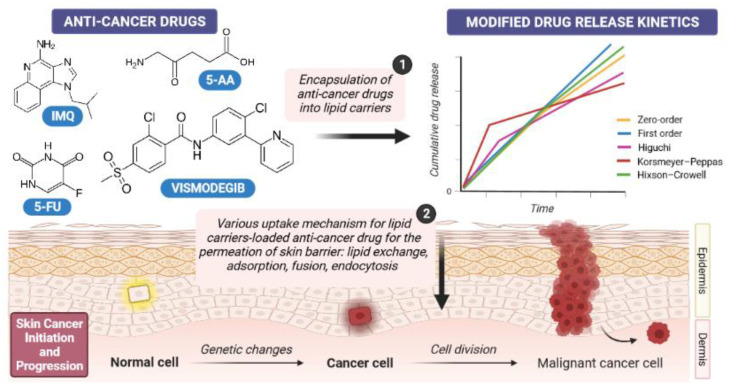
Scheme of targeted anticancer drug delivery using lipid nanocarriers (1), modified drug release kinetics, and various uptake mechanisms for skin barrier permeation (2). Below, a malignant cancer cell progression is shown (own drawing).

For the treatment of skin cancers, several types of nanoparticles, such as polymeric, lipid and metal-based nanoparticles, have been proposed to load a range of chemically differentiated anticancer drugs (Figure 2). Among them, lipid nanoparticles (e.g., solid lipid nanoparticles (SLNs), nanostructured lipid carriers (NLCs), liposomes, and oil-in-water (o/w) nanoemulsions) (Figure 3) are particularly interesting, as they can be synthesized from lipid materials that comprise the skin (e.g., fatty acids; waxes; mono-, di-, and tri-glycerides; and phospholipids), offering great biocompatibility and biodegradability [55,56,57]. Lipid nanoparticles can also be surface-functionalized with aptamers for site-specific targeting [58,59] and show a modified-release profile [60], reducing the risk of toxicity (Figure 4).

Most effective nanoparticles usually have a mean diameter between 1 and 100 nm, although the size of nanoparticles generally ranges from 1 to 1000 nm, as described by Zielińska et al. (2021) [61]. The solid core of lipid nanoparticles offers several benefits, namely loading of hydrophobic/poorly soluble drugs to improve their solubility in vivo, the protection of loaded drugs against degradation in a physiological environment, and controlled release via a targeted approach. Nanoparticles can be coated with ligands for site-specific targeting to certain receptors to reduce the risk of systemic distribution of anticancer drugs and thus reduce the toxicity of the treatment [61].

The skin is the largest organ of the human body and acts as a protective barrier against the permeation of xenobiotics. Therefore, specific strategies to overcome this barrier are needed [53,54]. The use of anticancer drugs targeted via lipid nanoparticles is an advanced therapeutic approach to increase the bioavailability of these drugs through the skin. Due to the compatibility and easily degradable lipids used in their production, lipid nanoparticles are well-tolerated, are devoid of toxicity, and can be applied for topical, dermal, and transdermal delivery of drugs. Souto et al. (2021) discussed several mechanisms of permeation/penetration of elastic/ultra-deformable liposomes and of lipid nanoparticles through the skin [53].

Depending on the type of lipid carrier and loaded drug, different release profiles (e.g., zero-order, first-order, Higuchi, Korsmeyer–Peppas, and Hixson–Crowell) can be obtained [62]. The lipophilic character of proposed carriers enables more than 80% of the drug to be loaded into the lipid matrices. The release profile of drug-loaded lipid nanoparticles is commonly evaluated over the course of 24 h. After this time, a varying amount (calculated percent) of the drug can be released. As shown in Figure 4, the release kinetics of anticancer drug-loaded lipid nanoparticle formulations can be investigated by fitting the resulted release data to the equations of different models, namely, zero-order, first-order, Higuchi, Korsmeyer–Peppas, and Hixson–Crowell [58,63]. Due to the empirical character of these equations, the release kinetics can be investigated through nonlinear dynamics in the form of logistic type laws [63].

Aptamers are short DNA or RNA single-started sequences that can bind to specific ligand molecules with high specificity and lack immunogenicity [64]. Conjugation of aptamers with nanoparticles has become an up-and-coming technique for therapy and tumor imaging. In particular, AS1411, an aptamer that specifically binds to nucleolin, a protein highly expressed only in cancer cells, has been proposed, as well as conjugation with lipid nanoparticles [65].

Regarding the risk of toxicity, the administration route of nanoparticles and time of exposure are the primary concerns. If nanoparticles reach the blood, they can accumulate in different locations, such as the liver, spleen, kidneys, or even the brain [66]. The toxicological profile of nanoparticles is governed not only by their chemical composition but also by physical parameters, such as surface area, size, surface charge, and aggregation state [67]. After cellular uptake upon, e.g., endocytosis, nanoparticles can be stored in cellular compartments for months [68]. Moreover, the risk of chronic toxicity with daily topical use of nanoparticles is still unknown [69,70]. The use of lipids with biodegradable properties is encouraged to design nanoparticles with a potential non-toxic profile [71]. Even the approved liposomal formulation containing doxorubicin was reported to cause skin disorders [72]. Recent studies have also shown an increased rate of squamous cell carcinoma in patients treated with liposomal doxorubicin for an extended period. It is unknown whether the reason for squamous cell carcinoma is doxorubicin, liposomal form, or both [73].

Oral vismodegib treatment is a gold standard for locally advanced and metastatic BCC; however, its limitations are associated with toxic side effects. The transdermal route is among the possible mechanisms of direct drug delivery to cancer lesions. To increase the permeation of vismodegib, ultradeformable liposomes obtained from phosphatidylcholine and sodium cholate, which can penetrate the stratum corneum, were evaluated [74]. The capacity for drug skin penetration was evaluated by comparing vismodegib in DSMO solution and vismodegib loaded in liposomes, showing about seven times increased penetration after using the liposomal formulation 1 h after administration (*p* < 0.0005). The longer the incubation time, the higher the amount of vismodegib that penetrated the skin up to 8 h (*p* < 0.02). The daily routine oral dose of vismodegib is 150 mg, with a drug bioavailability of about 31.8%, so the concentration of the drug was around 3 µg/mL. Considering that the volume of the skin disc containing the liposomes was about 0.64 cm^3^ and after 8 h of incubation 5.4 µg of vismodegib concentration, Calienni et al. reported the concentration of vismodegib as about 8.4 µg/mL and thrice higher in comparison to orally administered vismodegib. The use of nanoparticles could therefore directly improve vismodegib delivery to BCC lesions with higher drug concentration and lower risk of toxic effects [74].

Imiquimod is one of the most effective drugs for the treatment of nodular BCC but has limitations due to cutaneous permeation [75]. It stimulates the immune system to release cytokines and causes inflammation, which leads to apoptosis of cancer cells. To improve skin penetration, microneedles (polyvinylpyrrolidone-co-vinyl acetate) loaded with imiquimod were studied. Skin cross sections of imiquimod alone or with microneedles were described. Nanoparticles loaded with imiquimod were localized intradermally, whereas imiquimod alone was found only in the stratum corneum [75].

The use of topical 5-FU is sometimes a non-surgical option for BCC patients. SLN composed of stearic acid and lecithin were produced to load 5-fluorouracil for the treatment of skin carcinoma [76]. Several semi-solid formulations (e.g., from sodium carboxymethyl cellulose, hydroxypropyl methyl cellulose (HPMC), and chitosan) were then developed for topical administration of the chemotherapeutic drug in vivo. To confirm the efficiency of SLN for topical delivery of the hydrophilic chemotherapeutic drug, in vivo studies in tumor-bearing male balb/C mice were conducted, confirming the increased the penetration and enhanced efficacy of 5-FU. The authors described a marked decrease in inflammatory reaction and small hemorrhagic areas compared to both positive and drug-free controls. The in vivo results were consistent with the in vitro diffusion outputs using the semi-solid formulations. The sodium carboxymethyl cellulose semi-solid achieved the best diffusion and improved the release profile in comparison to HPMC and chitosan-composed gel matrices.

Ginseng is a plant traditionally used in oriental medicine with confirmed immunomodulatory, anti-inflammatory, and antioxidative activity and has been proposed as preventive approach against cancer. Nanoparticles with *Panax quinquefolium* (American ginseng) root extract were used in SKH1 hairless mice to prevent skin damage and carcinogenesis [77]. In one group, UVB irradiation was performed after application of extract-loaded nanoparticles, whereas the second group of nanoparticles was administered after UVB irradiation. Histopathological examination of the skin and analysis of blood were performed, resulting in an improved effect of the nanoparticles pre-treatment against UVB radiation, with lower blood cytokine (TNF-α and IL-1β) serum levels and limited pathological changes in the skin. Compared to UVB-irradiated control mice, TNF–α was 64% and, for IL-1β, 56% lower in a group with *Panax quinquefolium* prevention. The group receiving nanoparticles after UVB radiation did not show relevant changes in cytokine levels compared to the control group [77].

Photodynamic therapy (PDT) with 5-aminolaevulinic acid (5-AA) is an option for superficial BCC treatment, although it has limitations in terms of 5-AA penetrating deep layers of skin. Navarro-Triviño et al. published an article about a 7.8% 5-AA nanoemulsion-based gel (NBG). The 7.8% 5-AA NBG was previously found to trigger two to five times higher protoporphyrin IX formation in the epidermis than 20% 5-AA [78]. A total of 31 patients with superficial BCC were enrolled in the study. The patients were first treated with 5-AA NBG. After incubation, the remaining gel was wiped off, and the lesions were illuminated with a red-light LED lamp. All patients received two sessions of PDT one week apart. According to dermatoscopic, if there was a remaining lesion, another course of PDT with 5-AA NBG was carried. Efficacy was evaluated 3, 6, and 12 months after the last PDT treatment; 13 patients were identified as ‘responders’ after two PDT 5-AA NBG courses, and ten patients were identified as ‘responders’ after more than two PDT 5-AA NBG courses. One patient was reported as a non-responder after two cycles of PDT 5-AA NBG, and seven patients were so identified after more than two cycles of PDT 5-AA NBG. Complete clearance response rates were statistically significantly lower in patients who received more than two cycles of PDT 5-AA NBG (*p* = 0.045). All responding patients after 3 months also had a complete response after 6 and 12 months [79].

**Figure 5:**
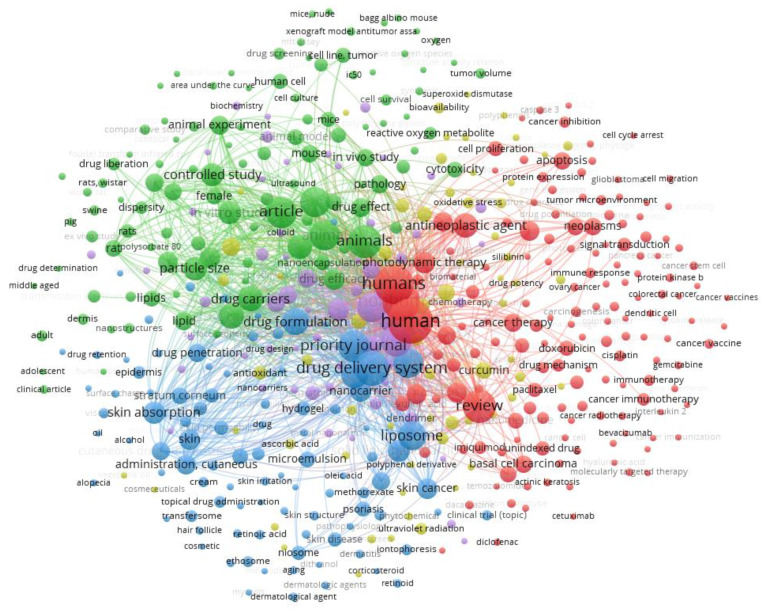
Bibliometric map generated by VOSviewer software [83] using “basal cell carcinoma” and “lipid nanoparticles” as keywords (from the Scopus database).

There are some limitations of nanoparticles used in the topical treatment of BCC, including the potentially high cost of this novel type of treatment, as only selected and specialized laboratories can make such exceptional drug delivery systems. There is also still a limited number of papers reporting on the use of nanoparticles in BCC patients. Some pivotal trials with cell lines and draft mice models have also been described, whereas melanoma and squamous cell carcinoma are the most commonly described targets for nanoparticle treatment [80,81,82]. A search on the Scopus database in May 2022 yielded a total of 327 papers reporting on any aspects related to basal cell carcinoma and lipid nanoparticles (Figure 5), highlighting the use of nanoparticles for drug delivery for topical administration of chemically different drugs (e.g., doxorubicin, paclitaxel, imiquimod, polyphenol derivatives, cetuximab, bevacizumab, diclofenac, retinoid, and corticosteroids).

Eczematous reactions are the most frequently reported side effects associated with the use of nanoparticles for the topical treatment of BCC, which may discourage patients from continuing treatment, even if existing drug formulations have similar side effects. Increased penetration through the stratum corneum may also lead to the accumulation of nanoparticle material if it is not biodegradable (e.g., gold) in human organs and veins. The mechanism by which nanoparticles may influence the well-being of BCC patients is still unknow. According to the clinicaltrials.gov (accessed on 22 March 2022) website, there are 354 clinical trials registered in BCC patients, although none involves nanoparticles.

## 5. Conclusions

The development of nanoparticles loading chemotherapeutic drugs for anticancer treatment is the future of non-melanoma cancer therapy, as these delivery systems possess a unique capacity to penetrate deeper into skin layers upon topical application. The selection of nanoparticle type is instrumental in reaching the site of action with limited toxic effects. Therefore, lipid nanoparticles are of considerable interest for old and frail patients or patients with high aesthetic expectations, given the biocompatibility and biodegradability of such systems. Further trials with cell lines, animal models, and BCC patients are highly encouraged in order to expand knowledge on the use of these particles for the treatment of BCC.
